# Retraction: MicroRNA-140-5p inhibits cell proliferation, migration and promotes cell apoptosis in gastric cancer through the negative regulation of THY1-mediated Notch signaling

**DOI:** 10.1042/BSR-2018-1434_RET

**Published:** 2023-01-19

**Keywords:** Gastric cancer, microRNA-140-5p, Migration, Notch signaling pathway, Proliferation, THY1

This Retraction follows an Expression of Concern relating to this article previously published by Portland Press. This article is being retracted from *Bioscience Reports* at the request of the Editor-in-Chief and the Editorial Board following receipt of a notification from a reader, alerting the Editorial Board to what appear to be duplicate regions between two panels of Figure 7B; specifically, between the miR-140-5p inhibitor panel and the miR-140-5p inhibitor+si-Notch1 panel. The Editorial Office has also identified similar regions in two panels of Figure S3, between the miR-140-5p inhibitor panel and the si-NC panel. There are additional concerns regarding the western blots from figures 2, 3, 5, 9, S1 and S5. The authors have been contacted with regards to the retraction and have not responded to the Journal's queries or the concerns raised. Given the extent of the issues raised, the Editorial Board stand by the decision to retract the article.

